# Hofmeister effect of anions on calcium translocation by sarcoplasmic reticulum Ca^2+^-ATPase

**DOI:** 10.1038/srep14282

**Published:** 2015-10-05

**Authors:** Francesco Tadini-Buoninsegni, Maria Rosa Moncelli, Niccolò Peruzzi, Barry W. Ninham, Luigi Dei, Pierandrea Lo Nostro

**Affiliations:** 1 Department of Chemistry “Ugo Schiff”, University of Florence, 50019 Sesto Fiorentino (Firenze), Italy; 2 CSGI, University of Florence, 50019 Sesto Fiorentino (Firenze), Italy; 3 Research School of Physical Sciences and Engineering, Australian National University, Canberra, Australia 0200

## Abstract

The occurrence of Hofmeister (specific ion) effects in various membrane-related physiological processes is well documented. For example the effect of anions on the transport activity of the ion pump Na^+^, K^+^-ATPase has been investigated. Here we report on specific anion effects on the ATP-dependent Ca^2+^ translocation by the sarcoplasmic reticulum Ca^2+^-ATPase (SERCA). Current measurements following ATP concentration jumps on SERCA-containing vesicles adsorbed on solid supported membranes were carried out in the presence of different potassium salts. We found that monovalent anions strongly interfere with ATP-induced Ca^2+^ translocation by SERCA, according to their increasing chaotropicity in the Hofmeister series. On the contrary, a significant increase in Ca^2+^ translocation was observed in the presence of sulphate. We suggest that the anions can affect the conformational transition between the phosphorylated intermediates E_1_P and E_2_P of the SERCA cycle. In particular, the stabilization of the E_1_P conformation by chaotropic anions seems to be related to their adsorption at the enzyme/water and/or at the membrane/water interface, while the more kosmotropic species affect SERCA conformation and functionality by modifying the hydration layers of the enzyme.

Hofmeister, or specific ion effects are ubiquitous. They occur in simple bulk solutions and at interfaces, in water and in non-aqueous solutions, and consist in the different effect that salts exert in a particular system^1^,^2^. For example the viscosity^3^ and the pH^4^ of electrolyte solutions, the swelling capacity of a gelator^5^, and the activity of an enzyme^6^ are examples of phenomena in which specific ion effects take place. Usually they emerge when the salt concentration is above 0.1 M, *i.e.* when purely electrostatic models (*e.g.* the Debye-Hückel or the DLVO theories) do not work any more^1^, but in some case they can be observed even in the micromolar range^7^,^8^.

The occurrence of specific ion effects in biochemistry is very well documented by an enormous number of papers^1^,^2^, that followed the pioneering studies of Franz Hofmeister on egg yolk proteins^9^. However a consistent physico-chemical framework that can predict the effect of a particular electrolyte in a given system is still missing. Some groups have proposed different hypotheses for the mechanism that controls Hofmeister phenomena. The different hydration properties of the single ions^10^,^11^, the salt-induced modification of the water structure^3^, the onset of non-electrostatic interactions at moderate and high salt concentrations^1^, and the polarity and charge of surfaces^12^ are the most significant features in whose terms specific ion effects are currently being interpreted. More recently, a significant effort has been made in order to reproduce specific ion effects in simulation studies^13^,^14^, and in order to take into account ionic dispersion forces in the calculation and prediction of activity and osmotic coefficients^15^.

The aim of the present research is to investigate specific ion effects on the pumping activity of the sarcoplasmic reticulum Ca^2+^-ATPase (SERCA). SERCA is a well characterized cation transport ATPase^16^,^17^,^18^,^19^ that is obtained with vesicular fragments of sarcoplasmic reticulum (SR). SERCA couples the hydrolysis of one molecule of ATP to the active transport of two Ca^2+^ ions from the cytoplasm to the SR lumen, thus inducing muscle relaxation. It is now well established that the Ca^2+^ pump also countertransports two to three protons and that the transport cycle is electrogenic^20^,^21^,^22^,^23^.

According to the E_1_-E_2_ scheme (Fig. 1) derived from the original reaction diagram of de Meis and Vianna^24^, the activation of SERCA requires the binding of two Ca^2+^ per enzyme molecule (E_1_Ca_2_), followed by phosphorylation by ATP and formation of a phosphoenzyme intermediate (E_1_P). The free energy derived from ATP is used by the phosphoenzyme for a conformational transition (E_1_P to E_2_P) that favours the translocation and release of the bound Ca^2+^ against its concentration gradient. Ca^2+^ ions are delivered to the intravesicular lumen in exchange for lumenal protons, that are translocated across the membrane to the cytosolic side during the following enzyme dephosphorylation. Hydrolytic cleavage of the phosphoenzyme is the final step, which allows the enzyme to undergo a new transport cycle.

It is known that various anions are able to affect partial and overall enzymatic reactions of the electrogenic ion pump Na^+^, K^+^-ATPase. The interaction of anions with Na^+^, K^+^-ATPase was characterized by biochemical and equilibrium fluorescence measurements, kinetic experiments using fluorescence dyes and electrical measurements on bilayer lipid membranes^25^,^26^,^27^,^28^,^29^,^30^,^31^. The order of anion effectiveness in influencing the Na^+^, K^+^-ATPase kinetics and transport activity follows the Hofmeister series. On the other hand, only a few studies on the interaction of anions with SERCA were reported. It was shown that certain anions can affect Ca^2+^ and H^+^ gradients produced by SERCA in reconstituted proteoliposomes^32^, as well as calcium uptake, ATPase activity and nucleotide binding in native SR membranes^33^.

To investigate the effects of several anions on the ATP-dependent Ca^2+^ translocation by SERCA, we employed an electrical method, which makes use of a solid supported membrane (SSM), depicted in Fig. 2. This technique has extensively been used to investigate the ion translocation mechanism of the SERCA pump^34^,^35^,^36^,^37^,^38^,^39^,^40^. The SSM represents a model system for a bilayer lipid membrane with the advantage of being mechanically so stable that solutions can be rapidly exchanged at the surface. In a typical SSM experiment, SR vesicles containing SERCA are adsorbed at the SSM surface and exposed to a concentration jump of a suitable substrate, *e.g.* Ca^2+^ or ATP^35^. By rapidly changing from a solution containing no substrate for the protein to one that contains a substrate, the protein can be activated and an electrical current is detected, which is related to the displacement of positive charge, *i.e.* Ca^2+^ ions, within the protein^35^,^36^.

In this contribution we report on the current measurements following ATP concentration jumps on SERCA-containing vesicles adsorbed on SSMs, in the presence of different potassium salts. We detected a specific ion effect on the ATP-induced Ca^2+^ translocation, and discuss the results in terms of the conformational transition between the phosphorylated intermediates E_1_P and E_2_P of the SERCA cycle.

## Results and Discussion

We performed current measurements on native SR vesicles containing SERCA adsorbed on a SSM, in order to investigate the effect of different potassium salts on the ATP-dependent Ca^2+^ translocation by SERCA, keeping the cation (K^+^) constant. The effect of the different anions was compared to that of chloride, which represents the reference anion.

A 100 μM ATP concentration jump was carried out in the presence of 10 μM free Ca^2+^, 1 mM Mg^2+^ and 100 mM potassium salt at pH 7. For each selected anion, the corresponding ATP-induced current transient was compared to that measured in the presence of 100 mM KCl, taken as a control measurement (Fig. 3). The charge obtained by numerical integration of the ATP-induced current transient is due to an electrogenic event corresponding to the translocation and release of bound Ca^2+^ upon phosphorylation by ATP^35^,^36^,^41^ within the first enzyme cycle.

The current transients are shown in Fig. 3, and the corresponding charges, obtained by numerical integration and normalized with respect to the charge determined in the presence of 100 mM KCl, are reported in Table 1. In the presence of the monovalent chaotropic anions, *i.e.* NO_3_^−^, Br^−^, SCN^−^ and I^−^, we observed a remarkable reduction in the ATP-induced current amplitude compared to the current measured in the presence of KCl (Fig. 3). The order of effectiveness of the monovalent anions in reducing the ATP-dependent charge movement is I^−^>SCN^−^>Br^−^>NO_3_^−^ (Table 1). The reduction in the translocated charge correlates well with the position of these anions in the direct Hofmeister series^1^.

To confirm the occurrence of a Hofmeister phenomenon, we plotted Q_norm_ as a function of the lyotropic number N (see Fig. 4). This parameter was introduced by Voet in his studies on the lyotropicity of salt-induced precipitation of starch^42^ and represents a sort of fingerprint of Hofmeister phenomena^43^.

We recall here that in the case of Na^+^, K^+^-ATPase-containing membrane fragments adsorbed on bilayer lipid membranes the amount of Na^+^-related charge transferred by the protein following an ATP concentration jump in the presence of 150 mM bromide or iodide was reduced to about 40% with respect to the charge obtained in the control measurement (150 mM NaCl), whereas a slight increase in the translocated charge was observed in the presence of 300 mM chloride^31^. The and Hasselbach also reported a 50% inhibition of ATP-dependent calcium uptake in native SR vesicles in the presence of 150 mM thiocyanate or 200 mM nitrate^33^. Interestingly, in the presence of the kosmotropic SO_4_^2−^ anion (100 mM K_2_SO_4_) we observed a significant increase in the current amplitude (and related charge) with respect to the current detected in the presence of 100 mM KCl (Fig. 3 and Table 1). In the case of 100 mM K_2_SO_4_ the ionic strength of the electrolyte solution is three times higher than that of a 100 mM solution of a 1:1 salt. In order to check whether the higher ionic strength of the electrolyte solution may contribute to increase the current amplitude, we performed 100 μM ATP concentration jumps on SERCA using KCl solutions of increasing ionic strength. No significant effect on the ATP-induced current transient in the presence of 200 mM KCl was detected, and a slight decrease in the translocated charge with 300 mM KCl (data not shown), thereby confirming that the increase in current amplitude and associated charge in the presence of sulphate can be ascribed to the specific nature of the SO_4_^2−^ anion and not to the different ionic strength of the solution.

In order to investigate the mechanism through which the different anions affect the translocated charge in a specific manner, we checked the correlation between the values of Q_norm_ and some of the most important ionic physico-chemical parameters, that account for ion specificity.

Figures 5, 6 and 7 show the value of Q_norm_ as a function of the anion static polarizability (α), of the surface tension molar increment (σ) and of the hydration entropy (ΔS_hydr_), respectively. These parameters reflect the different nature of the ions. The former represents the softness of the electron cloud and contributes in setting the extent of the frequency-dependent dispersion forces (see eq. 14 in ref. 1). While electrostatic interactions—that are not ion specific—dominates at low salt concentration (*e.g.* below 10^−3^ M), non-electrostatic forces emerge when the concentration of the ions is rather large (usually higher than 10^−2^ M) and directly depend on the nature of the single ion^1^. Kosmotropic species possess small values of α, while chaotropic are more polarizable and therefore may establish non-electrostatic dispersion forces with other molecules and ions. The plot in Fig. 5 suggests that the more polarizable ions (iodide and thiocyanate) interact more strongly with the colloidal species and induce a greater effect on the translocated charge.

Figures 6a,b show the variation of Q_norm_ with the air/water surface tension molar increment and with the lysozyme/water surface tension molar increment, respectively. This parameter is defined as σ = ∂Δγ/∂c, and measures the effect of each ion on the air/water surface tension change (Δγ) as a function of the salt concentration (c)^1^. Kosmotropes possess large values of σ.

One may argue that in our study ions do not interact with an air/water interface, but rather with a membrane/water or with an enzyme/water interface. For this reason we listed in Table 1 the values of σ_LW_, *i.e.* the surface tension molar increment at the lysozyme/water interface^44^. Figure 6b shows the plot of Q_norm_ versus σ_LW_. In fact this protein/water interface is certainly closer to the system studied in the present paper. In any case, the trends shown in Figure 6 confirm that the chaotropic species produce a significant lowering in the value of Q_norm_, as a result of the ion adsorption at the membrane/water or at the enzyme/water interface, and therefore suggests that the specific interfacial adsorption of the ions is involved in the mechanism that modifies the pumping activity of SERCA, depending on the nature of the background electrolyte.

Figure 7 shows the plot of Q_norm_ as a function of the ion hydration entropy. ΔS_hydr_ reflects the ability of an ion to polarize the water molecules that reside in the first hydration shell around the enzyme and/or the membrane^45^. This plot suggests that sulphate has a strong polarizing effect and therefore weakens the water-enzyme interactions and eventually partly dehydrate the macromolecule. Interestingly, this effect is less pronounced for the chaotropic species (from nitrate to iodide), as these cannot significantly modify the extent and strength of the hydration layer around SERCA, but rather specifically adsorb at its interface, as indicated in Fig. 6.

Before proceeding with the discussion of the charge translocation data, we point out that no specific ion effect was detected when the binding of Ca^2+^ ions to SERCA was carried out without ATP. In particular, 10 μM free Ca^2+^ concentration jumps were performed and the corresponding current signals were recorded. The charge obtained by numerical integration of the Ca^2+^-induced current transient is due to the electrogenic event related to binding of Ca^2+^ ions to SERCA from the cytoplasmic side in the absence of ATP^35^,^36^,^37^. The charge determined for a typical chaotropic (NO_3_^−^) and for a typical kosmotropic (SO_4_^2−^) anion, at a concentration of 100 mM, are 0.86 ± 0.02 and 0.97 ± 0.11, respectively. These charges are normalized to the value obtained in the presence of 100 mM KCl (control measurement).

**Figure 1:**
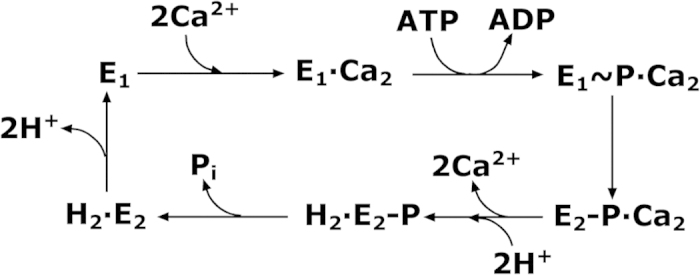
Schematic diagram of sequential reactions in the transport cycle of SERCA.

The results indicate, within the experimental error, that the displaced charge is not significantly affected by the presence of 100 mM nitrate or sulphate, thereby suggesting that the two anions do not interfere with the binding of cytoplasmic Ca^2+^ to the ATPase transport sites, *i.e.* the E_1_ → E_1_·Ca_2_ step in the diagram depicted in Fig. 1.

We may tentatively explain the effect of the various anions on the ATP-induced Ca^2+^ translocation by SERCA in terms of the conformational transition between the phosphorylated intermediates E_1_P and E_2_P of SERCA, *i.e.* the E_1_P → E_2_P transition (see Fig. 1). In fact, in the case of Na^+^, K^+^-ATPase it was shown that kosmotropic anions favour the E_2_P conformation whereas chaotropic anions favour the E_1_P conformation^25^,^26^. Thus, if the E_2_P conformation of SERCA is destabilized in favour of the E_1_P conformation in the presence of chaotropic anions, such condition will interfere with the electrogenic release of bound Ca^2+^ ions, thereby leading to a reduced charge translocation (Fig. 3 and Table 1). A similar explanation for the effect of bromide and iodide on ATP-dependent sodium translocation by Na^+^, K^+^-ATPase was proposed^31^. On the other hand, if the E_2_P conformation of SERCA is stabilized in the presence of the kosmotropic sulphate, lumenal release of Ca^2+^ ions is favoured and an increase in the amount of the translocated charge is expected (Fig. 3 and Table 1). Such an effect of the anions on the translocated charge is not observed in Ca^2+^-concentration jump experiments in the absence of ATP, *i.e.* when the E_1_ → E_1_·Ca_2_ step is investigated, consistent with the assumption that the anions can interfere with the E_1_P → E_2_P transition of the SERCA cycle. However, we cannot completely rule out the possibility that the net positive charge traslocated by SERCA could in principle be partially reduced if any passive diffusion of anions in the same direction occurs across the membrane. In particular sulphate, which is expected to have a lower membrane permeability than chloride, could compensate the active translocation of positive charge by SERCA to a lesser degree than chloride and lead to a higher overall charge being transferred.

We speculate that the stabilization of the E_1_P conformation of the SERCA pump by chaotropic anions is related to adsorption of these anions on the surface of the SR vesicles, as indicated by Figs 5 and 6. In experiments on ion interaction with the lipid surface of a SSM membrane, Fendler and co-workers demonstrated that chaotropic anions and kosmotropic cations are attracted to the membrane regardless of its composition^46^. It is also worth mentioning the work by Clarke and Lüpfert, who proposed that binding of chaotropic anions to phosphatidylcholine liposomes may cause a reduction of the intramembrane dipole potential^47^. We now suggest that the adsorption of chaotropic anions on the SR vesicle surface may bring about the stabilization the E_1_P conformation of the enzyme and hence interfere with the translocation and release of calcium ions at the lumenal side of the SR vesicle. We further speculate that a local perturbation of the membrane curvature may assist and promote the enzyme conformational change (see Fig. 8), according to a mechanism proposed by Larsson^48^ and in agreement with recent results on the Na^+^, K^+^-ATPase^49^, in which a local deformation of the lipid membrane was suggested to occur simultaneously with conformational changes of the sodium pump.

Finally, in the presence of the monovalent anions, particularly in the case of the most chaotropic I^−^ and SCN^−^, a component of negative amplitude is observed in the current trace (Fig. 3). Such a current component of rather small amplitude decays to zero with a time constant of ~100 ms, which is determined by the resistive and capacitive elements of the equivalent circuit model describing the SSM with adsorbed membrane entities^34^,^50^,^51^. In particular, this negative current component has previously been attributed to a backflow of charge through membrane fragments incorporating Na^+^, K^+^-ATPase^50^ and SR vesicles containing SERCA^34^ adsorbed on SSMs. It was shown that the negative current component was more pronounced under experimental conditions preventing the ion pump from completing the transport cycle, *i.e.* when the stationary pump current is suppressed. We may thus conclude that the negative current component observed in the presence of the chaotropic anions is due to their hindering effect on the completion of the SERCA transport cycle, through the stabilization of the E_1_P conformation.

## Conclusions

We report on the current measurements following ATP concentration jumps on sarcoplasmic reticulum Ca^2+^-ATPase (SERCA)-containing vesicles adsorbed on solid supported membranes (SSM), in the presence of different potassium salts. We detected a significant specific ion effect on the ATP-induced calcium translocation, and discuss the results in terms of the specific adsorption and of the hydration properties of the anions at the interface between the enzyme or the vesicle and the aqueous surrounding medium.

These results seem to indicate that the effect of the anions is related to their ability to stabilize either of the two phosphorylated intermediates (E_1_P and E_2_P) and modify the performance of the enzyme. For the chaotropes the main effect is due to their adsorption at the enzyme/water interface, while the more kosmotropic species (sulfate and chloride) act in perturbing the hydration layers of the enzyme and therefore in modifying its conformation and functionality.

Eventually, the adsorption of chaotropic ions or the partial de-hydration operated by kosmotropes at the enzyme surface may lead to a conformational state change between the phosphorylated intermediates of the SERCA cycle that affects the translocation of Ca^2+^ ions across the enzyme.

## Methods

### ATPase preparation

Native SR vesicles containing SERCA were obtained by extraction from the fast twitch hind leg muscle of New Zealand white rabbit, followed by homogenization and differential centrifugation, as described by Eletr and Inesi^52^. The vesicles so obtained (light vesicles), derived from longitudinal SR membrane, contained only negligible amounts of the ryanodine receptor Ca^2+^ channel associated with junctional SR. The total protein concentration, determined by the Lowry procedure^53^, was 8.4 mg/mL. SERCA content corresponds to ~50% of the total protein^16^.

### Measurement of charge movements

**Figure 2:**
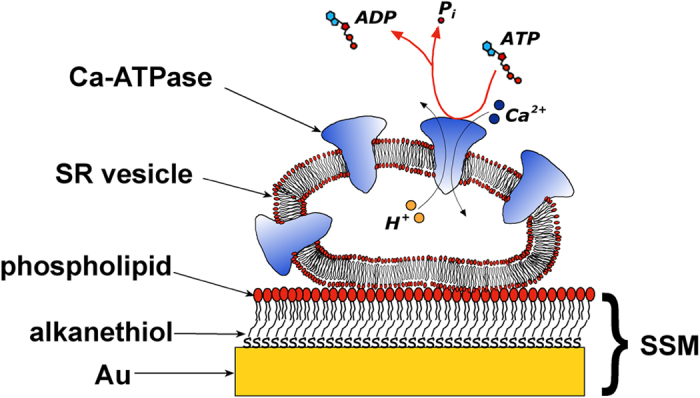
Schematic diagram of a SR vesicle adsorbed on a solid supported membrane (SSM) and subjected to ATP activation (not drawn to scale). For simplicity, only four Ca-ATPase molecules are shown in the vesicle. Adapted with permission from ref. 40. Copyright 2009 American Chemical Society.

**Figure 3:**
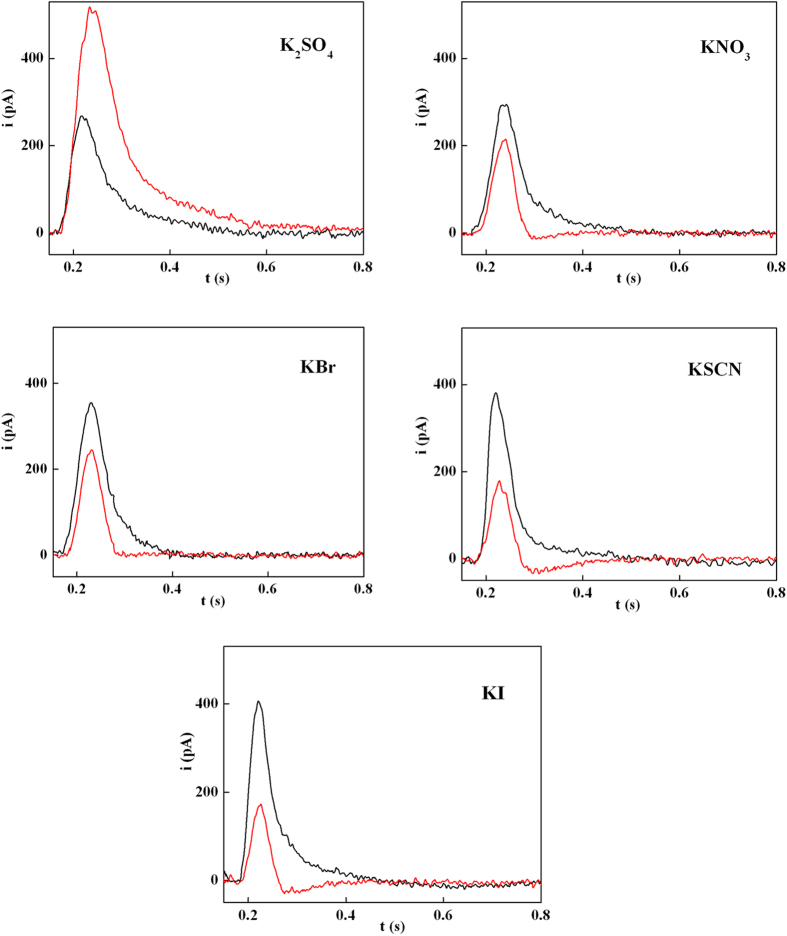
Current transients induced by 100 μM ATP concentration jumps in the presence of 10 μM free Ca^2+^ were recorded at 100 mM KCl (black curve, control measurement) or 100 mM potassium salt (red curve).

**Figure 4:**
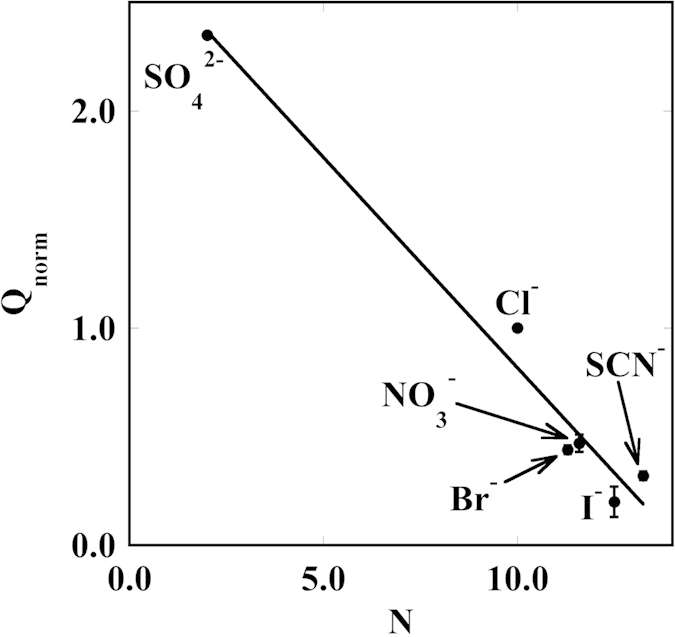
Q_norm_ as a function of the lyotropic number N. The line is a guide for the eye.

**Figure 5:**
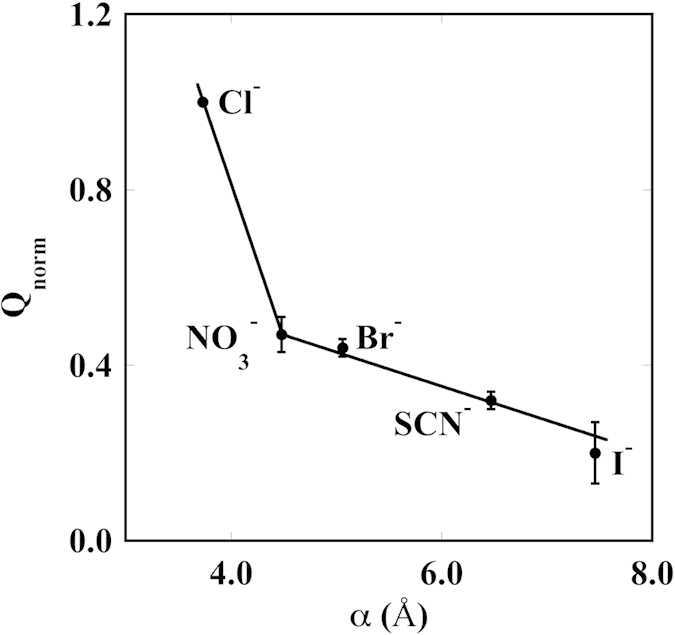
Q_norm_ versus the anion static polarizability (α). The lines are a guide for the eye.

**Figure 6:**
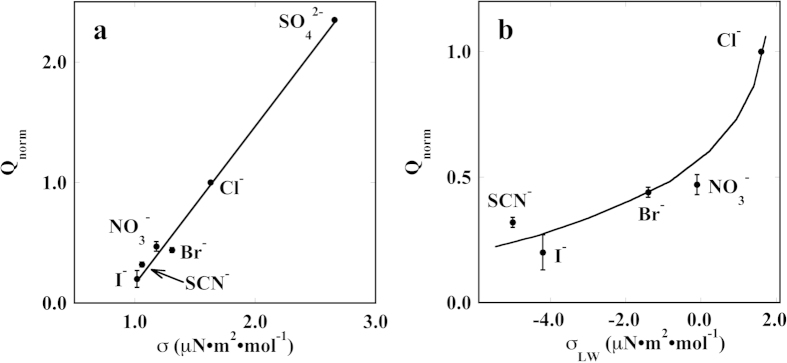
Q_norm_ versus the anion air/water surface tension molar increment defined as σ = ∂Δγ/∂c (a), and versus the lysozyme/water surface tension molar increment σ_LW_ (b). The lines are a guide for the eye.

**Figure 7:**
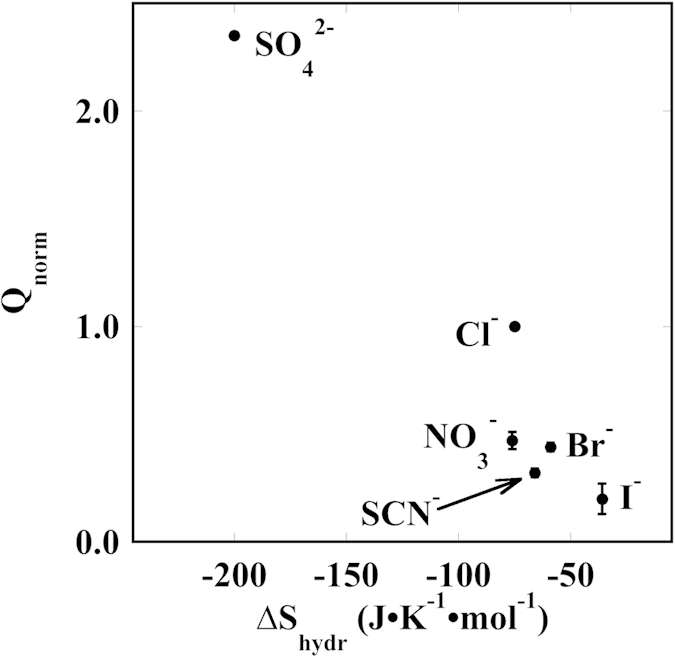
Q_norm_ versus the anion entropy of hydration (ΔS_hydr_).

**Figure 8:**
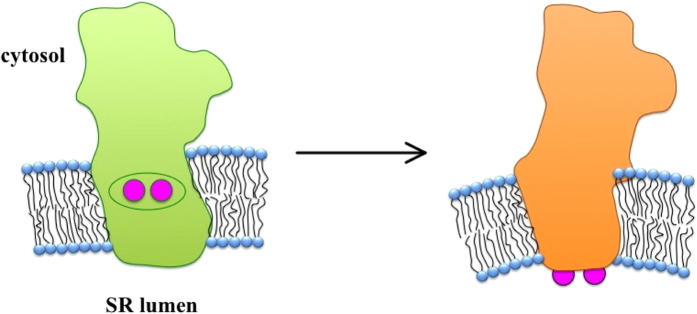
Idealized scheme depicting the conformational change E_1_P → E_2_P in SERCA, and the partial local perturbation of the membrane curvature. The purple circles represent the Ca^2+^ ions that are translocated from the cytoplasm (top) to the sarcoplasmic reticulum (SR) lumen (bottom). For the conformational change of the enzyme see Figure 14 in ref. 18 and Fig. 1 in ref. 55.

Charge movements were measured by adsorbing the SR vesicles containing SERCA onto a hybrid alkanethiol/phospholipid bilayer anchored to a gold electrode (SSM). The SSM consists of an octadecanethiol monolayer covalently bound to the gold surface via the sulphur atom, with a diphytanoylphosphatidylcholine monolayer on top of it, as illustrated in Fig. 2^34^,^50^.

SR vesicles, following a brief sonication, were adsorbed on the SSM surface during an incubation time of 60 min. After the adsorption, SERCA was activated by the rapid injection of a solution containing an appropriate substrate, *e.g.* Ca^2+^ or ATP. If at least one electrogenic step, *i.e.* a net charge movement across the vesicular membrane generated by the protein is involved in the relaxation process that follows the protein activation, then a current transient will be detected due to the capacitive coupling between the vesicle membrane and the SSM^35^,^36^,^51^. In particular, the electrical response of the ion pump is monitored under potentiostatic conditions. In this case, movement of a net charge across the activated protein is compensated by a flow of electrons along the external circuit to keep the applied voltage ΔV constant across the whole metal/solution interface. The resulting current transient is recorded as a function of time. Normally, experiments are carried out under short circuit-conditions, *i.e.* at zero applied voltage relative to the reference electrode. The numerically integrated current transient is related to a net charge movement within the protein, which depends upon the particular electrogenic step (*i.e.* following Ca^2+^ or ATP concentration jumps).

The effect of potassium salts on charge movements was investigated at a salt concentration of 100 mM, keeping constant the concentrations of the other solutes. In all experiments two buffered solutions were employed, the “non-activating” and the “activating” solution:

- In Ca^2+^ concentration-jump experiments, the non-activating solution contained 100 mM potassium salt, 10 mM 3-(N-morpholino)propansulfonic acid (MOPS, pH 7.0), 0.25 mM ethylene glycol tetraacetic acid (EGTA) and 1 mM MgCl_2_; the activating solution contained, in addition, 0.25 mM CaCl_2_ (10 μM free Ca^2+^).
- In ATP concentration-jump experiments, the non-activating solution contained 100 mM potassium salt, 10 mM MOPS (pH 7.0), 1 mM MgCl_2_, 0.25 mM EGTA and 0.25 mM CaCl_2_ (10 μM free Ca^2+^); the activating solution contained, in addition, 100 μM ATP.

Free Ca^2+^ concentration was calculated with the computer program WinMAXC^54^.

The concentration jump experiments were performed by the SURFE^2^R^One^ device (Nanion Technologies). The temperature was maintained at 22–23 °C for all the experiments.

To verify the reproducibility of the current transients on the same SSM, each single measurement was repeated 6 times and then averaged to improve the signal to noise ratio. Standard deviations did not exceed 5%. Moreover, each set of measurements was usually reproduced using two-three different SSM sensors.

## Additional Information

**How to cite this article**: Tadini-Buoninsegni, F. *et al.* Hofmeister effect of anions on calcium translocation by sarcoplasmic reticulum Ca^2+^-ATPase. *Sci. Rep.* **5**, 14282; doi: 10.1038/srep14282 (2015).

## Figures and Tables

**Table 1:** Normalized charges (Q_norm_) following 100 μM ATP concentration jumps in the presence of 10 μM free Ca^2+^ and different potassium salts (100 mM), lyotropic number (N), air/water surface tension molar increment (σ, in μN·m^2^·mol^−1^), lysozyme/water surface tension molar increment (σ_LW_, in μN·m^2^·mol^−1^), and entropy of hydration (ΔS_hydr_, in J·mol^−1^·K^−1^) for the single anion.

Anion (X)	Q_norm_	N^a^	σ^b^	σ_LW_^c^	ΔS_hydr_^b^
Cl^−^	1	10.0	1.63	1.6	−75
SO_4_^2−^	2.35 ± 0.01	2.0	2.66		−200
Br^−^	0.44 ± 0.02	11.3	1.31	−1.4	−59
NO_3_^−^	0.47 ± 0.04	11.6	1.18	0.1	−76
SCN^−^	0.32 ± 0.02	13.2	1.06	−5.0	−66
I^−^	0.20 ± 0.07	12.5	1.02	−4.2	−36

Charges are normalized with respect to the value obtained in the presence of 100 mM KCl (control measurement). Data represent the mean ± s.e.m. of three independent measurements.

^a^from ref. 43.

^b^from ref. 45.

^c^from ref. 44.
